# Comment on: Out-of-hospital cardiac arrest: the prospect of E‑CPR in the Maastricht region

**DOI:** 10.1007/s12471-016-0853-3

**Published:** 2016-05-18

**Authors:** M. T. Blom, S. G. Beesems, M. Hulleman

**Affiliations:** Academic Medical Center, University of Amsterdam, Amsterdam, the Netherlands

Dear Editors,

we read with great interest the study `Out-of-hospital cardiac arrest: the prospect of E‑CPR in the Maastricht region’ by Sharma, Pijls and colleagues, as recently published by the Netherlands Heart Journal [1]. The survival rates after out-of-hospital cardiac arrest (OHCA) presented in this study are impressive. There is a great need for Dutch data on outcome after OHCA, and we applaud the efforts of the authors to collect and present these data.

However, the authors compare their results (survival after OHCA in patients *who have been transported to hospital*) with other studies that have different inclusion criteria (*all* OHCA patients, including those who die at the scene and are not transported to hospital [2, 3]). Evidently, survival rates in patients who were transported to hospital will be much higher. While the authors refer to an editorial recently published in this journal discussing this exact issue [4], they still make an incorrect comparison. Furthermore, the authors compare proportion of favourable neurological outcome *in survivors* (presumably defined as modified ranking score 0), with proportion of favourable neurological outcome (defined as cerebral performance category) of *all OHCA patients in whom resuscitation was attempted*, and state that the difference may be explained by presence of a shockable rhythm and early return of spontaneous circulation. This is surprising.

It would be very interesting to compare data on OHCA and outcome of resuscitation efforts in the Netherlands, using uniform inclusion and outcome definitions as recommended by the Utstein criteria [5]. This would provide valuable insight into factors influencing outcome after OHCA in the Netherlands.
